# Improvement of the Mechanical Properties of 1022 Carbon Steel Coil by Using the Taguchi Method to Optimize Spheroidized Annealing Conditions

**DOI:** 10.3390/ma9080693

**Published:** 2016-08-12

**Authors:** Chih-Cheng Yang, Chang-Lun Liu

**Affiliations:** 1 Department of Mechanical and Automation Engineering, Kao Yuan University, Kaohsiung 82151, Taiwan; 2 Graduate School of Fasteners Industry Technology, Kao Yuan University, Kaohsiung 82151, Taiwan; ching0608s@yahoo.com.tw

**Keywords:** spheroidized annealing, formability, Taguchi method

## Abstract

Cold forging is often applied in the fastener industry. Wires in coil form are used as semi-finished products for the production of billets. This process usually requires preliminarily drawing wire coil in order to reduce the diameter of products. The wire usually has to be annealed to improve its cold formability. The quality of spheroidizing annealed wire affects the forming quality of screws. In the fastener industry, most companies use a subcritical process for spheroidized annealing. Various parameters affect the spheroidized annealing quality of steel wire, such as the spheroidized annealing temperature, prolonged heating time, furnace cooling time and flow rate of nitrogen (protective atmosphere). The effects of the spheroidized annealing parameters affect the quality characteristics of steel wire, such as the tensile strength and hardness. A series of experimental tests on AISI 1022 low carbon steel wire are carried out and the Taguchi method is used to obtain optimum spheroidized annealing conditions to improve the mechanical properties of steel wires for cold forming. The results show that the spheroidized annealing temperature and prolonged heating time have the greatest effect on the mechanical properties of steel wires. A comparison between the results obtained using the optimum spheroidizing conditions and the measures using the original settings shows the new spheroidizing parameter settings effectively improve the performance measures over their value at the original settings. The results presented in this paper could be used as a reference for wire manufacturers.

## 1. Introduction

A cold-heading-quality alloy steel rod is used to manufacture wire for cold heading. Generally, the wire made from the quality rod is spheroidizing annealed, either in a single process or after drawing the finished product [1]. The wire that is spheroidizing-annealed-in-process is produced by drawing wire coil into wire, followed by heat treatment, cleaning and coating, and then a final drawing operation for cold forming.

Spheroidization of cementite lamellae through spheroidized annealing improves the ductility of steel [2,3,4]. Rad-Con Inc. (Cleveland, OH, USA) provided a spheroidized annealing process that produced steel wire with little or no decarbonization under completely computerized control [2]. The majority of all spheroidizing activity is performed to improve the cold formability of steels. A spheroidized microstructure is desirable for cold forming because it lowers the flow stress of the material. Steels may be spheroidized to produce a structure of globular carbides in a ferritic matrix [5].

Many studies on the mechanisms and kinetics of spheroidization have been undertaken [6,7,8,9]. O’Brien and Hosford [6] investigated the spheroidization of medium carbon steels, AISI 1541 and AISI 4037, used in the bolt industry with two process cycles, the intercritical cycle and subcritical cycle. Introducing defects in the cementite by severe plastic deformation is one effective method to increase the spheroidization speed. Hono et al. [7] revealed that the cementites in near-eutectic steel can be spheroidized more easily after a severe drawing. Shin et al. [8] studied the enhanced spheroidization kinetics in terms of carbon dissolution from cementites and defects induced in cementites by severe plastic deformation, and revealed that an increase in accumulated strains in the equal channel angular pressed steel decreased the spheroidization temperature and time. Gul’ et al. [9] developed a new method for more intense spheroidization of cementite to accelerate spheroidization. Spheroidization is produced by non-isothermal holding at high temperatures, by means of an internal heat source.

In the bolt industry, most companies use a subcritical process for spheroidized annealing, by simply heating the products to below the lower critical temperature and maintaining this temperature. Some companies simply purchase steel wires, and cold reduce them, and spheroidize them before selling them to bolt manufacturers. Some companies, which manufacture bolts, spheroidize the wires themselves before cold heading. A cold-heading-quality AISI 1022 steel wire is usually used to manufacture self-drilling screws and tapping screws. The wire has to be spheroidizing annealed after drawing the wire coil (Φ5.5 mm) to a specific size with section-area reductions of about 60%.

The quality of spheroidized annealed wire affects the forming quality of the screws. Various parameters affect the quality of spheroidized annealing, such as the spheroidized annealing temperature, prolonged heating time, furnace cooling time and flow rate of nitrogen (protective atmosphere). The effects of the spheroidized annealing parameters affect the quality characteristics of wires, such as the tensile strength and hardness.

The Taguchi method is a quality improvement technique that uses experimental design methods for efficient characterization of a product or process, combined with a statistical analysis of its variability with the fact that pre-production experiments, properly designed and analyzed, can significantly contribute to efforts towards the accurate characterization and optimization of industrial processes, the quality improvement of products, and the reduction of costs and waste [10]. In this study, the Taguchi method is used to optimize spheroidized annealing conditions to improve the mechanical properties of AISI 1022 low carbon steel wire.

## 2. Experiment Design

AISI 1022 low carbon steel wire is investigated in this study. Its chemical composition is listed in Table 1. A subcritical process is used for spheroidized annealing of the steel wire, simply heating it to below the lower critical temperature and maintaining this temperature.

Four process parameters with three levels, as listed in Table 2, are chosen as the experimental factors in this study. Every factor has three levels to spheroidize wire in order to evaluate the mechanical properties of the wire. The parameters of Level 2 are the original spheroidized annealing process conditions, which was using in the company.

The Taguchi method allows the changing of many factors at the same time in a systematic way, ensuring the reliable and independent study of the factors’ effects. The orthogonal array table, L_9_(3^4^), is used as an experimental design for these four factors [11], as listed in Table 3.

In this study, two quality characteristics of the spheroidized annealing wire, tensile strength and hardness, are investigated. Each test trial, including 10 specimens, is followed by each fabrication process and the results are then transformed to the S/N ratio (signal-to-noise ratio). Spheroidizing provides the needed ductility for cold heading. Through spheroidized annealing, the ductility of steel wire may be improved, and the hardness, which is obtained from the Vickers hardness test, may be reduced as well. Therefore, in terms of the desired characteristics for the hardness, the smaller the better, and the S/N ratio is [11]

(1) $$\text{S}/\text{N}=-10\cdot \;\log(\;\mu^{2}+\;S^{2})$$

where μ is the mean of each trial and *S* is the standard deviation.

When the hardness is reduced to improve the ductility of the steel wire through spheroidized annealing, the strength of the steel wire is simultaneously decreased. However, a given strength of the annealing steel wire has to be provided for cold heading. Therefore, the tensile strength of the steel wire is the main quality characteristic, with a target value of 383 MPa, which is assigned by the company. The S/N ratio for the nominal-the-best response is [11]

(2) $$\text{S}/\text{N}=-10\cdot \log[{\left(\;\mu -m\;\right)}^{2}+\;S^{2}]$$

where μ is the mean of each trial, *m* is the target value, and *S* is the standard deviation. The tensile tests are conducted on a 30 ton universal testing machine under a constant ram speed of 25 mm/min at room temperature. The dimensions of the tensile specimen are Φ3.5 mm × L300 mm.

Analysis of variance (ANOVA) is an effective method to determine the significant factors and the optimal fabrication conditions to obtain optimal quality. For the Taguchi method, the experimental error is evaluated with ANOVA to carry out the significance test of the various factors. The nature of the interaction between factors is considered as experimental error [11]. If the effect of a factor in comparison to the experimental error is sufficiently large, it is identified as a significant factor. The confidence level of a factor is evaluated with the experimental error to identify the significant factor that influences the material property of the spheroidized annealing wire.

## 3. Results and Discussion

**Figure 1:**
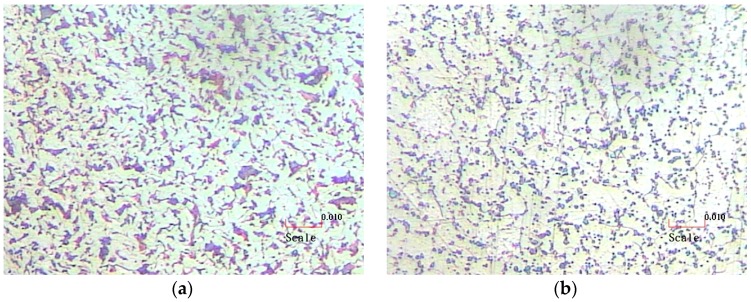
Microstructures of drawn AISI 1022 steel wires (×500). (**a**) Non-spheroidized; (**b**) Spheroidized.

The microstructure of drawn steel wire is shown in Figure 1a. This is not yet spheroidized annealed. The tensile strength and hardness are, respectively, about 822 MPa and 285 HV due to heavy plastic work. Spheroidizing is the process of producing a microstructure in which the cementite is in a spheroidal distribution, as shown in Figure 1b. The globular structure obtained gives improved formability to the steel wire. When the wire is fabricated following the original spheroidized annealing process conditions (Level 2 in Table 2), the mean tensile strength and mean hardness are 388.7 MPa and 141.3 HV, respectively. They are about half of the non-spheroidized wire.

The optimum experimental results of the tensile strength and hardness (mean, μ; standard deviation, *S*; and S/N ratio) of spheroidized annealed steel wire are shown in Table 4 and Table 5 respectively. The mean tensile strength varies from 368.9 to 407.0 MPa. The mean tensile strengths of tests L2, L3 and L7 are smaller than the target value, as shown in Table 4. The mean tensile strength of test L8 is very close to the target value and its standard deviation is the smallest of the nine tests.

As shown in Table 5, the mean hardness varies from 134.0 to 147.7 HV, and the mean values of tests L2, L3 and L8 are smaller than the value at the original settings. The properties of spheroidized annealed steel wire are obviously altered with various spheroidized annealing process conditions.

### 3.1. Tensile Strength

To obtain optimum quality, analysis of variance (ANOVA) is an effective method to determine significant factors and optimum fabrication conditions. The contribution and confidence level of each factor constructed in Table 6 could identify the significant factor affecting the tensile strength of wire. The contribution of a factor is the percentage of the sum of squares (SS), that is, the percentage of the factor variance to the total quality loss [10,11]. The effect of a factor may be pooled to error if its confidence level or contribution is relatively small. It is clear from the ANOVA table that the contribution of the spheroidized annealing temperature (A) is 87.0% of the total variation, which is the highest contributor to the variability of the experimental results. The contribution of prolonged heating time (D) is 10.2%, which is the second highest contribution. However, the factors of the furnace cooling time (C) and the flow rate of nitrogen (B) are not significant for the S/N ratio because their contributions are relatively small. With the pooling of errors from the non-significant factors (B and C), the error estimation for the S/N ratio is obtained [11] and then the confidence levels are 99.9% and 95.4%, respectively, for the spheroidized annealing temperature (A) and prolonged heating time (D). That is, both factors, particularly the spheroidized annealing temperature, significantly affect the tensile strength of the steel wire, with more than a 95.0% confidence level.

**Figure 2:**
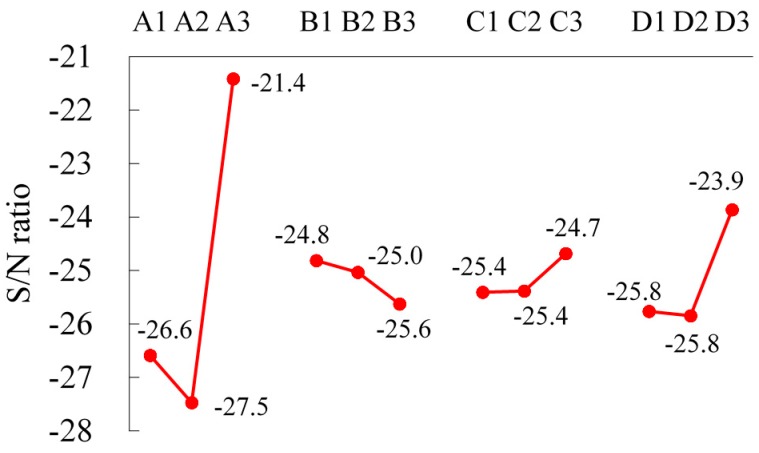
The factor response diagram for tensile strength.

Figure 2 illustrates the factor response diagram and the level averages of four factors with respect to the S/N ratio. For each factor, the effect is the range of the level averages and the maximum level average is the optimum level [10,11]. It is obviously revealed that, for the four factors, the original levels (Level 2) are not the optimum fabricating parameters to obtain the target tensile strength. For the significant factors of the spheroidized annealing temperature (A) and prolonged heating time (D), Level 3 for the spheroidized annealing temperature (705 °C, A3) and Level 3 for the prolonged heating time (8 h, D3) are evidently the optimum levels, as shown in Figure 2. However, it is observed that their responses are not linear either with the annealing temperature or prolonged heating time. For the spheroidized annealing temperature, the optimum level is closer to the lower critical temperature. Its response is much more effective than the other two levels.

The effects of the other two factors, the flow rate of nitrogen (B) and furnace cooling time (C), are relatively small. The optimum levels are Level 1 for the flow rate of nitrogen (5 Nm^3^/h, B1) and Level 3 for the furnace cooling time (8.5 h, C3), respectively.

### 3.2. Hardness

For the hardness of the annealed steel wire, the ANOVA table of the S/N ratio is constructed in Table 7. It is evident from Table 7 that the highest contributors to the variability of the experimental results are the flow rate of nitrogen (B, 58.5%), the prolonged heating time (D, 21.5%) and the spheroidized annealing temperature (A, 18.8%). However, the furnace cooling time (C) is not a significant factor because its contribution is relatively small. With the pooling of errorsfrom the non-significant factor (C), the confidence levels are 93.7%, 97.9% and 94.5%, respectively, for the spheroidized annealing temperature (A), flow rate of nitrogen (B) and prolonged heating time (D). That is, the hardness of the steel wire is significantly affected by the spheroidized annealing temperature, flow rate of nitrogen and prolonged heating time, with more than a 90.0% confidence level.

**Figure 3:**
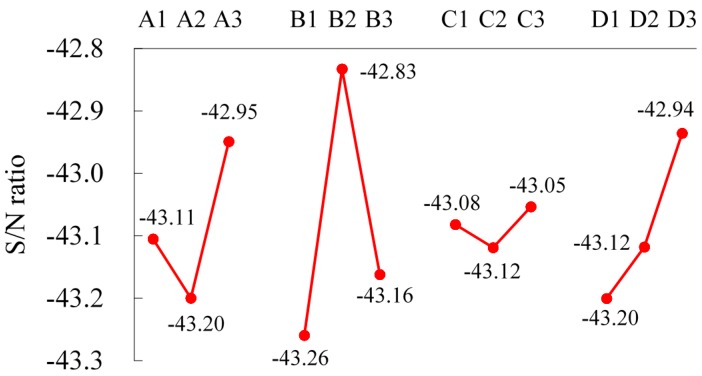
The factor response diagram for hardness.

The factor response diagram and the level averages of four factors with respect to the S/N ratio are illustrated in Figure 3. It is observed that the responses are not linear either with the annealing temperature, the flow rate of nitrogen or the furnace cooling time; but are linear with the prolonged heating time. It is revealed that increasing the prolonged heating time may lower the hardness of the wire. For the factor of the flow rate of nitrogen, which is a protective atmosphere to prevent decarbonization while heating the wire, although it is the most significant factor in this study, its effect is even smaller than the effect on tensile strength and it is not a significant factor for tensile strength, as shown in Figure 2.

For the three significant factors of the spheroidized annealing temperature (A), flow rate of nitrogen (B) and prolonged heating time (D), the optimum levels are Level 3 for the spheroidized annealing temperature (705 °C, A3), Level 2 for the flow rate of nitrogen (10 Nm^3^/h, B2) and Level 3 for the prolonged heating time (8 h, D3), respectively, as shown in Figure 3. The effect of the furnace cooling time (C) is relatively small. Level 3 is the optimum level for the furnace cooling time (8.5 h, C3).

With the optimum analysis for the quality characteristics of tensile strength and hardness, the optimum conditions are shown in Table 8. The factors of spheroidized annealing temperature (A) and prolonged heating time (D) are obviously significant for both the tensile strength and hardness and with the same level. Therefore, the optimum levels are determined as Level 3 for the spheroidized annealing temperature (705 °C, A3) and Level 3 for the prolonged heating time (8 h, D3). The factor of the flow rate of nitrogen (B) is not significant for tensile strength, but is significant for hardness. Thus, Level 2 for the flow rate of nitrogen (10 Nm^3^/h, B2) is then chosen as the optimum level. The factor of furnace cooling time (C) is not significant either for tensile strength or hardness. The original level, Level 2, for the furnace cooling time (8 h, C2) is determined.

### 3.3. Confirmatory Experiments

**Figure 4:**
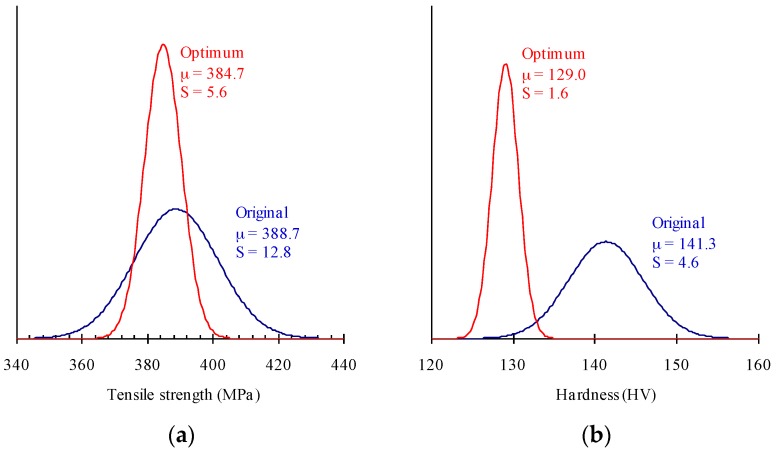
The probability distribution diagram for (**a**) tensile strength; (**b**) hardness.

In order to verify the predicted results, wire is fabricated using the optimum levels: A3, B2, C2 and D3 (as described in Table 8). Figure 4 shows the original (using level 2s in Table 2) and optimal probability distributions, respectively, for the tensile strength and hardness of the steel wire. Compared with the original results, the optimum mean tensile strength of 384.7 MPa is not only closer to the target value, but also the deviation is decreased by about 56%. The optimum mean hardness of 129.0 HV is considerably decreased compared to the original mean hardness of 141.3 HV. In addition, the deviation decreases by about 65% compared to the original result. The new parameter settings evidently improve the performance measures, such as ductility and strength, over their value at the original settings, and thus the quality of the spheroidized annealed steel wire. Therefore, the formability of the AISI 1022 steel wire is effectively improved.

## 4. Materials and Methods

**Figure 5:**
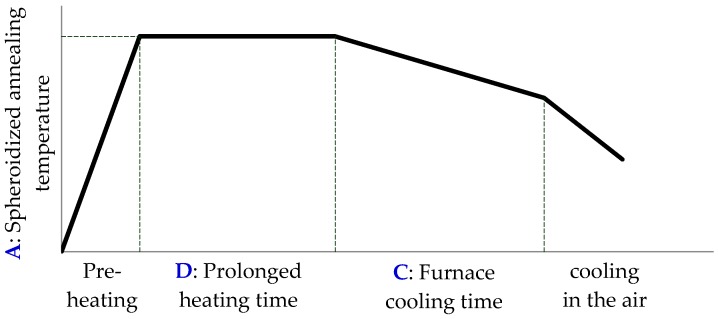
The spheroidized annealing procedure.

In this study, the wire is spheroidizing annealed after drawing AISI 1022 steel wire coil (Φ5.5 mm) to a specific size (Φ3.5 mm) with section-area reductions of about 60%. The steel wire coil is manufactured (Φ5.5 mm, Al-killed) by China Steel Corporation, Kaohsiung, Taiwan. Its chemical composition is listed in Table 1. The steel wire is spheroidizing annealed, procedures as shown in Figure 5, with CCP-2820 pit type annealing furnace (Tainan Chin Chang Electrical Co., Ltd., Tainan, Taiwan). The Taguchi method allows the changing of many factors at the same time in a systematic way. The orthogonal array table, L_9_(3^4^), is used as an experimental design for the factors [11], as listed in Table 3.

## 5. Conclusions

The alloy steel rod with cold-heading quality is usually used for the manufacture of wire for cold heading. Generally, the wire made from the quality rod is spheroidized annealed. The majority of spheroidizing activity is performed to improve the cold formability of steel wires. The quality of spheroidized annealed steel wire affects the forming quality of screws. In this study, the Taguchi method is used to obtain optimum spheroidized annealing conditions to improve the mechanical properties of AISI 1022 low carbon steel wire. The spheroidized annealing qualities of steel wire are affected by various factors, such as the spheroidized annealing temperature, prolonged heating time, furnace cooling time and flow rate of nitrogen. The effects of the spheroidized annealing conditions affect the quality characteristics of steel wire, such as the tensile strength and hardness. Since a given strength of the annealing steel wire has to be provided for cold heading, the tensile strength is the main quality characteristic of spheroidized annealed steel wire, with a target value of 383 MPa. It is experimentally revealed that the spheroidized annealing temperature (A) and the prolonged heating time (D) are the significant factors; the determined levels are Level 3 for the spheroidized annealing temperature (705 °C, A3), Level 3 for the prolonged heating time (8 h, D3), Level2 for the furnace cooling time (8 h, C2), and Level 2 for the flow rate of nitrogen (10 Nm^3^/h, B2). In addition, the optimum mean tensile strength is 384.7 MPa, and the optimum mean hardness is 129.0 HV. The new spheroidizing parameter settings evidently improve the performance measures over their values at the original settings. The formability of the AISI 1022 steel wire is effectively improved. The results may be used as a reference for wire manufacturers.

## Figures and Tables

**Table 1:** Chemical composition of AISI 1022 low carbon steel wires (wt. %).

C	Mn	P	S	Si	Ni	Cr	Cu	Al
0.22	0.76–0.77	0.012–0.013	0.002–0.008	0.02–0.04	0.01–0.03	0.04	0.01–0.08	0.030–0.034

**Table 2:** Experimental factors and their levels for L_9_ orthogonal array.

Factor		Level 1	Level 2	Level 3
A:	Spheroidized annealing temperature (°C)	695	700	705
B:	Flow rate of nitrogen (Nm^3^/h)	5	10	15
C:	Furnace cooling time (h)	7.5	8.0	8.5
D:	Prolonged heating time (h)	7	7.5	8

**Table 3:** L_9_(3^4^) orthogonal array experimental parameter assignment.

Exp. No.	A: Spheroidized Annealing Temperature (°C)	B: Flow Rate of Nitrogen (Nm^3^/h)	C: Furnace Cooling Time (h)	D: Prolonged Heating Time (h)
L1	695	5	7.5	7.0
L2	695	10	8.0	7.5
L3	695	15	8.5	8.0
L4	700	5	8.0	8.0
L5	700	10	8.5	7.0
L6	700	15	7.5	7.5
L7	705	5	8.5	7.5
L8	705	10	7.5	8.0
L9	705	15	8.0	7.0

**Table 4:** The experimental results for tensile strength.

Exp. No. ^a^	T1	T2	T3	T4	T5	T6	T7	T8	T9	T10	μ (MPa)	*S*	S/N Ratio
L1	417	409	362	387	417	407	403	398	400	376	397.6	16.96	−27.10
L2	365	370	374	359	380	387	398	340	382	335	368.9	18.93	−27.38
L3	411	379	381	365	367	360	365	367	372	354	372.0	15.02	−25.29
L4	388	415	409	413	398	366	385	403	400	390	396.8	14.18	−26.06
L5	400	373	385	386	414	382	397	412	409	432	398.9	17.16	−27.48
L6	381	415	427	411	417	413	407	413	398	388	407.0	13.24	−28.88
L7	377	369	375	391	356	374	381	377	387	394	378.1	10.68	−21.28
L8	376	384	388	387	393	401	399	393	391	378	389.1	8.00	−20.25
L9	394	386	374	392	382	412	400	392	378	398	390.8	10.90	−22.71

^a^ Experimental conditions as defined in Table 3.

**Table 5:** The experimental results for hardness.

Exp. No. ^a^	T1	T2	T3	T4	T5	T6	T7	T8	T9	T10	μ (HV)	*S*	S/N Ratio
L1	147	145	147	148	148	144	152	154	155	137	147.7	4.98	−43.4
L2	153	136	135	145	151	137	133	137	136	135	139.8	6.81	−42.9
L3	138	136	144	146	145	144	135	149	142	133	141.2	5.08	−43.0
L4	148	145	148	143	147	150	148	146	137	143	145.5	3.56	−43.3
L5	141	132	144	140	142	161	139	142	137	138	141.6	7.20	−43.0
L6	141	147	149	143	147	143	148	144	146	155	146.3	3.77	−43.3
L7	151	141	147	144	137	143	142	154	135	138	143.2	5.76	−43.1
L8	138	133	135	131	139	134	138	133	126	133	134.0	3.66	−42.5
L9	146	147	148	146	145	136	148	144	142	139	144.1	3.78	−43.2

^a^ Experimental conditions as defined in Table 3.

**Table 6:** Variance analysis table of signal-to-noise (S/N) ratio for tensile strength.

**Factor**	**SS**		**DOF**		**Var.**	**Contribution**	
A	64.27		2		32.13	87.0%	
B	1.06		2		0.53	1.4%	
C	1.02		2		0.51	1.4%	
D	7.54		2		3.77	10.2%	
Total	73.88		8		--	100.0%	
Pooling of errors							
**Factor**	**SS**	**DOF**		**Var.**	**F**	**Confidence**	**Significance**
A	64.27	2		32.13	62.05	99.9%	Yes
B	Pooled						
C	Pooled						
D	7.54	2		3.77	7.28	95.4%	Yes
Error	2.07	4		0.52	S_exp_ = 0.72		
Total	73.88	8		* At least 95.0% confidence level			

SS: sum of squares; DOF: degree of freedom; Var.: variance; F: F-ratio; S_exp_: experimental error.

**Table 7:** Variance analysis table of signal-to-noise (S/N) ratio for hardness.

**Factor**	**SS**	**DOF**	**Var.**	**Contribution**		
A	0.096	2	0.048	18.8%		
B	0.300	2	0.150	58.5%		
C	0.006	2	0.003	1.3%		
D	0.110	2	0.055	21.5%		
Total	0.513	8		100.0%		
Pooling of errors						
**Factor**	**SS**	**DOF**	**Var.**	**F**	**Confidence**	**Significance**
A	0.096	2	0.048	14.96	93.7%	Yes
B	0.300	2	0.150	46.63	97.9%	Yes
C	Pooled					
D	0.110	2	0.055	17.10	94.5%	Yes
Error	0.006	2	0.003	S_exp_ = 0.06		
Total	0.513	8	* At least 90.0% confidence level			

SS: sum of squares; DOF: degree of freedom; Var.: variance; F: F-ratio; S_exp_: experimental error.

**Table 8:** Optimum condition table for spheroidized annealing.

Factor	Tensile Strength	Hardness	Optimum
A	A3 *	A3 *	A3
B	B1	B2 *	B2
C	C3	C3	C2
D	D3 *	D3 *	D3

* Significant factor.
